# Light Alcohol Consumption Does Not Protect Cognitive Function: A Longitudinal Prospective Study

**DOI:** 10.3389/fnagi.2018.00081

**Published:** 2018-03-26

**Authors:** Linda B. Hassing

**Affiliations:** Department of Psychology, University of Gothenburg, Gothenburg, Sweden

**Keywords:** cognitive aging, memory, alcohol consumption, abstainer bias, longitudinal study

## Abstract

Studies show that light to moderate alcohol consumption is related to better health and higher cognitive performance. However, it has been suggested that this association is caused by a systematic bias in the control group as many people abstain from drinking or quit because of health issues. Therefore, the group of non-drinkers is biased towards poor health and may not be suitable as a control group. The present study examined the effect of alcohol on cognitive performance while addressing this bias by excluding the non-drinkers. Thus, instead of comparing different levels of alcohol consumption to a non-drinking control group, a dose-response association was calculated between all levels of alcohol intake and cognitive performance. The study used information from a sample of people in the Swedish Twin Registry, who in their midlife (1967) participated in a survey on alcohol intake and 25 years later participated in a longitudinal study on cognitive aging (*N* = 486). The cognitive aging study took place on five occasions, at 2-year intervals, and included the Mini Mental State Examination (MMSE), tests of episodic memory, semantic memory and spatial ability. The association between midlife alcohol consumption and later cognitive performance was analyzed using growth curve models, adjusting for background variables. The findings showed that there was a significant negative dose-response association between alcohol intake in midlife and the MMSE, and the tests of episodic memory, such that higher intake in midlife was related to lower performance in old age. The associations between alcohol and semantic memory, and spatial ability respectively, were not significant. In contrast to findings from other studies, which show that low to moderate alcohol intake promotes cognitive function, the current study showed that alcohol intake was related to lower cognitive performance in a dose-response manner, even at low levels. The results from this study indicate that the observed benefits of moderate alcohol intake for cognitive function reported by others might be solely due to comparisons to an inappropriate control group, a group that is biased towards poor health. Hence, it is concluded that light alcohol intake may not protect cognitive function.

## Introduction

Many studies converge on the conclusion that long-term low to moderate alcohol intake protects against dementia and cognitive aging (for review, see Peters et al., 2008; Anstey et al., 2009). At the same time it is well documented that the acute effect of alcohol impairs cognitive performances such as attention, psychomotor speed, tracking ability, working memory and cognitive flexibility (Matthews and Silvers, 2004; Mintzer, 2007; Fillmore, 2011; Dry et al., 2012). It is somewhat paradoxical that the immediate effect of alcohol is detrimental whereas the long-term effect is thought to be protective. There is a broad consensus in the literature concerning the acute negative effect of alcohol on cognitive performance, however, there is an ongoing debate concerning the positive effect of long-term low to moderate alcohol intake (Naimi et al., 2005; Stockwell et al., 2012, 2016; Fekjær, 2013; Chikritzhs et al., 2015). The aim of this study was to examine the effect of long-term light alcohol consumption in relation to cognitive aging.

In general, studies show that there is a J-shaped association between alcohol consumption and a range of health outcomes, reflecting lower risk for low to moderate drinkers in comparison to abstainers, and higher risk for heavy drinkers in comparison to abstainers (for reviews, see Hill, 2005; Ronksley et al., 2011). The same pattern of findings is seen concerning the outcomes of dementia and cognitive functioning (Leroi et al., 2002; Ruitenberg et al., 2002; Truelsen et al., 2002; Mukamal et al., 2003; Ganguli et al., 2005; Stampfer et al., 2005; Deng et al., 2006; Peters et al., 2008; Stott et al., 2008; Anstey et al., 2009; Weyerer et al., 2011; Kesse-Guyot et al., 2012; Almeida et al., 2014; Nooyens et al., 2014). However, the validity of the positive health-effect of alcohol, has been questioned recently (Naimi et al., 2005; Fekjær, 2013; Chikritzhs et al., 2015; Stockwell et al., 2016). For example, in a critical analysis of the literature, Fekjaer presented an extensive list of diseases and medical conditions that have been shown to have a J-shaped association with alcohol intake, although in many cases a causal association seems highly unlikely (i.e., common cold, hearing loss, and negative child development). Other researchers point to methodological shortcomings of observational studies, one major critique being misclassification of abstainers. It has been known for a long time that the group of alcohol abstainers is biased towards poor health (Shaper et al., 1988; Fillmore et al., 2006, 2007a,b, 2008). This bias arises because the most common reason to abstain from drinking in late adulthood is poor health. Further, many people who do not drink are former drinkers who have quit for various other reasons. Hence, the control group typically consists of individuals who have poorer health in general, and includes individuals who have been exposed to alcohol earlier in life. A recent systematic review and meta-analysis of studies investigating alcohol use and mortality risk showed that after adjustment for abstainer biases no significant reduction in mortality risk was observed for low-volume drinkers (Stockwell et al., 2016).

The association between alcohol intake and cognitive function has not yet been tested while properly taking care of the abstainer bias. Given that the prevalence of ill health increases with aging, the impact of the abstainer bias is probably more pronounced in studies of older cohorts. Thus, it is possible that the protective effect of light alcohol intake on cognitive performance is only a spurious effect, a result of a comparison to a biased control group. The most common way to adjust for differences in health has been through statistical control. However, this may not be an efficient solution as information on health and diseases are often lacking or incomplete. The aim of the present work was to test the effect of long-term light alcohol intake on cognitive performance while accounting for the abstainer bias. To do this, a dose response association between alcohol intake in midlife and cognitive function 25 years later was estimated while controlling for the abstainer bias through exclusion of non-drinkers. Alcohol consumption reported in midlife was used because it is assumed that midlife alcohol intake better reflects life-time alcohol habits as compared to alcohol intake reported in old age. In line with the findings of Stockwell et al. (2016), it is expected that there will be no positive effect of light alcohol intake on cognitive performance when the abstainer bias has been accounted for.

## Materials and Methods

### Study Design and Data Sources

This study is based on data from two studies that have been linked; The Swedish Twin Registry and the OCTO-Twin study. The Swedish Twin Registry was established in the late fifties to study smoking and alcohol consumption in relation to health risks (Lichtenstein et al., 2002, 2006). In 1967 the participants in the Swedish Twin Registry answered a survey including detailed questions about alcohol consumption. The OCTO-Twin study started between 1991 and 1993 (McClearn et al., 1997) and included the same individuals who participated in the survey in 1967. The OCTO-Twin study was a longitudinal study on cognitive aging, including extensive in-person cognitive testing every other year for five test occasions. For the purpose of the present study, the survey data on alcohol intake from the Swedish Twin Registry in 1967 has been linked to the OCTO-Twin study data on cognitive test performance.

### Participants

A sample of 702 individuals, in 351 pairs, aged 80 years and older, was drawn from the oldest cohort (born 1901–1911) of the Swedish Twin Registry. The representativeness of the sample was supported by a study which compared one randomly chosen member of each dyad to a population-based sample of Swedish singletons of the same age (Simmons et al., 1997). The following individuals were excluded: (1) those with missing information on alcohol intake in the survey in 1967 (*n* = 113); (2) those with missing cognitive data (*n* = 47); and (3) those having dementia diagnosis at the first wave of the cognitive testing in 1991–1993 (*n* = 56). The total sample at the first wave of the longitudinal study included 486 individuals (175 men and 311 women). For a description of the sample attrition across the five waves see Table 1. The study was approved by the ethics committee of the Karolinska Institute, Stockholm, Sweden (Dnr. 89:98, 98–380) and by the Swedish Twin Registry. All participants were informed about the study. During the years of OCTO-Twin data collection written consent was not required. All participants were informed about the study in accordance with the ethics committee of the Karolinska Institutet, Sweden, the Swedish Data Inspection Board, and the institutional board at the Pennsylvania State University, USA.

**Table 1:** Sample size and attrition across the five waves.

	Wave 1 1991–1993	Wave 2 1993–1995	Wave 3 1995–1997	Wave 4 1997–1999	Wave 5 1999–2001
Participation, *n*	486	418	339	256	188
Reason for attrition					
Death, *n*		43	66	71	64
Refusal, *n*		21	12	10	3
Other reason, *n*		4	1	2	1

### Measures

#### Alcohol Consumption

**Figure 1:**
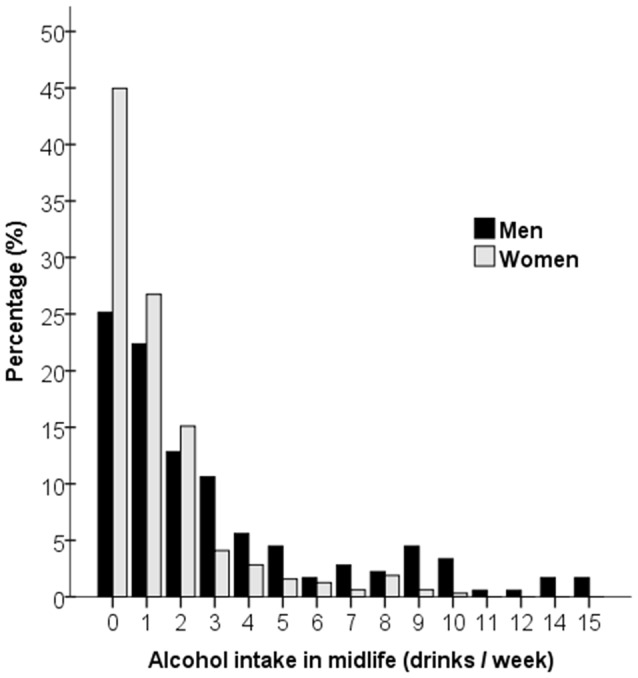
Alcohol intake in drinks per week in midlife for men and women.

The questions on alcohol consumption included in the survey in 1967 concerned if the participants used alcohol or not, which type of beverages they consumed (beer, wine, strong wine, or hard liquor), how often they drank alcohol and how much they drank on a typical occasion. Based on the complete information a total score was computed which estimated intake in grams of alcohol/week. The score was then converted into units/week, where 1 unit of alcohol corresponded to 12 g, or one drink, according to standards taking into account alcohol volume and amount. Because of low alcohol consumption in this cohort no distinction was made between types of beverages. Further, it was apparent that those who reported drinking alcohol, in most cases drank more than one type of beverage. There were no heavy alcohol users in this sample; the highest reported alcohol consumption was 15 units/week (see Figure 1).

#### Cognitive Tests

The longitudinal design encompassed five measurement occasions at 2-year intervals beginning in 1991–1993. Four cognitive tasks were selected to tap crystallized (semantic memory) and fluid abilities (episodic memory and spatial ability) as well as the Mini-Mental State Examination (MMSE). The two episodic memory tasks reflecting long-term memory were the Prose Recall test (a Swedish version of the Logical Memory in the Wechsler Memory Scale; Wechsler, 1991) and The Thurstone’s Picture recognition test (Thurstone and Thurstone, 1949). The semantic memory task was the Swedish version of the Information Task (Jonsson and Molander, 1964). To measure spatial ability the Block Design test (Dureman and Sälde, 1959) was used. Global cognitive status was measured by MMSE (Folstein et al., 1975). The test battery has been described elsewhere (Hassing et al., 2004). For the ease of comparisons across tasks, all cognitive measures were transformed into a *t*-score metric (*M* = 50; SD = 10).

#### Covariates

Educational level was recorded as years of formal education. Socioeconomic position was classified as low, middle and high. Smoking status was coded as 0 for non-smoker and 1 for current or previous smoker. Body mass index (BMI) was based on information from the survey in 1967 and was calculated by dividing the weight in kilos by the height in square meters (kg/m^2^). Information on diabetes and vascular diseases (hypertension, myocardial infarction, congestive heart failure and stroke) reflects lifetime prevalence. This information was obtained through a detailed review of medical records by a physician who made the final diagnoses on the basis of: (a) medical records containing information dating back at least to 1985; (b) medicine use; and (c) self-reported information concerning health and diseases, reported in the longitudinal follow-ups. Diagnoses were classified and based on ICD-10 criteria (for more details for this procedure see elsewhere (Nilsson et al., 2002). If there was any notification of a disease or a history of self-report the person was coded as having the disease. A variable indicating vascular disease was constructed by counting any notification of hypertension, myocardial infarction, congestive heart failure, or stroke (range 0–4). Diabetes was a dichotomized as 0 (no diabetes) and 1 (diabetes).

### Statistical Analyses

#### Abstainer Bias

To verify that the alcohol abstainers differ in many aspects from those who drink alcohol, the sample was stratified into two groups based on alcohol consumption in 1967; the alcohol abstainers (*n* = 181), and those who reported drinking alcohol (*n* = 305). Group comparisons, comparing the abstainers to drinkers, were conducted using independent *t*-tests for continuous variables (age, education, BMI in 1967, and the cognitive tests in wave 1), and Chi-square tests for categorical variables (sex, socioeconomic position, smoking, diabetes, hypertension, myocardial infarction, congestive heart failure, stroke and dementia).

#### Effects of Alcohol Intake on Cognitive Performance

To test the long-term effects of light alcohol intake on cognitive performance a series of mixed effects growth models were conducted. These models analyzed the association between midlife alcohol intake (continuous variable) and later cognitive performance, and rate of decline across the five waves. To handle the abstainer bias all participants who reported no alcohol consumption in midlife were excluded from the analyses.

A growth curve modeling approach was used. Conceptually, growth curve analysis involves estimating within-individual regressions of performance on time and on expected predictors of these individual regression parameters (i.e., individual performance at baseline and change over time). The multilevel model was characterized by a fixed part which contains average effects for the intercept (initial status) and slope (rate of change over time) and a random part which contains individual differences (variance) in the intercept, slope, and the within person residual. The models were tested using the missing at random assumption for missing cognitive outcomes. A three-level linear growth model was composed of a level-1 component of individual outcomes over time, a level-2 component which models individual fixed and random effects of initial status and change over time (person-level covariates can be added at this level), and the level-3 component which models variance associated with twin-pair status. This was done to handle the fact that a twin sample was used to draw conclusions about non-twins in which higher intraclass correlations are expected for monozygotic than for dizygotic twins. Thus, a three-level structure was characterized by longitudinal measurements nested within individuals who were nested within groups (twin dyad).

Two sets of models (Model 1 and Model 2) were run that estimated the following; level of cognitive performance (the Intercept), cognitive decline across time (the Slope), the effect of alcohol intake on cognitive performance (Alcohol), and the additional cognitive decline across time related to alcohol intake (Slope × Alcohol). Model 1 adjusted for differences in demographic characteristics, that is, age, education, sex and socioeconomic position. In Model 2 further adjustments were made for differences in health-related factors, that is, BMI in midlife, diabetes, vascular diseases and smoking.

## Results

### Abstainer Bias

The sample characteristics are presented in Table 2. Concerning the abstainer bias, the group of alcohol abstainers differed significantly from the group of drinkers in several background characteristics and health conditions. The group of abstainers had; greater proportion of women ($\chi_{\left(1\right)}^{2}$ = 20.52), lower education (*t*_(484)_ = 4.67), lower SES ($\chi_{\left(1\right)}^{2}$ = 19.40), higher BMI in midlife (*t*_(482)_ = 2.42), and poorer lifetime health condition, reflected by higher prevalence of diabetes ($\chi_{\left(1\right)}^{2}$ = 11.17), and hypertension ($\chi_{\left(1\right)}^{2}$ = 9.87), however, the abstainers were less frequently smokers ($\chi_{\left(1\right)}^{2}$ = 61.48). Descriptive data for the cognitive tests at the first wave of the cognitive testing in 1991–1993 are also presented in Table 2. The alcohol abstainers performed at a lower level on all cognitive tests as compared to drinkers; MMSE (*t*_(484)_ = 4.52), picture memory (*t*_(358)_ = 2.02), prose recall (*t*_(358)_ = 3.82), semantic memory (*t*_(449)_ = 6.21), spatial ability (*t*_(394)_ = 1.92).

**Table 2:** Sample characteristics of the total sample and across Alcohol groups.

	Total sample (*N* = 486)	No alcohol intake (*n* = 181)	Alcohol intake (*n* = 305)	*p*-value
Alcohol units/week, *M ± SD*	1.5 ± 2.7	0	3.1 ± 2.9	<0.001
Age (at first wave), *M ± SD*	83.0 ± 2.6	83.1 ± 2.5	82.9 ± 2.6	0.542
Sex (Women), *n* (%)	311 (64)	139 (77)	172 (56)	<0.001
Education (years), *M ± SD*	7.3 ± 2.4	6.7 ± 1.7	7.7 ± 2.7	<0.000
Lowest SES group, *n* (%)	228 (47)	102 (57)	126 (41)	<0.001
Ever smoked, *n* (%)	196 (40)	32 (18)	164 (54)	<0.001
BMI in 1967, *M ± SD*	24.5 ± 2.7	24.8 ± 2.6	24.2 ± 2.8	0.016
Diabetes, *n* (%)	80 (17)	43 (24)	37 (12)	0.001
Hypertension, *n* (%)	229 (47)	102 (56)	127 (42)	0.002
Myocardial infarction, *n* (%)	102 (21)	33 (18)	69 (23)	0.250
Congestive heart failure, *n* (%)	125 (26)	50 (28)	75 (25)	0.459
Stroke, *n* (%)	101 (21)	35 (19)	66 (22)	0.545
Incident dementia, *n* (%)	94 (19)	42 (23)	52 (17)	0.097
MMSE, *M ± SD*	52.0 ± 7.2	50.4 ± 7.4	53.0 ± 5.1	<0.001
Picture recognition, *M ± SD*	51.7 ± 9.2	50.3 ± 9.5	52.4 ± 9.0	0.044
Prose recall, *M ± SD*	50.5 ± 9.5	48.2 ± 9.4	51.8 ± 9.3	0.001
Semantic memory, *M ± SD*	50.8 ± 9.4	47.4 ± 10.4	52.9 ± 8.1	<0.001
Visuospatial ability, *M ± SD*	50.2 ± 10.1	49.0 ± 9.6	51.0 ± 10.3	0.056

*Note. The p-values refer to t-tests (df = 358–484) for continuous variables and to chi-square tests (df = 1), contrasting the No alcohol group to the Alcohol group*.

### Alcohol Intake in Relation to Level of Cognitive Performance

**Figure 2:**
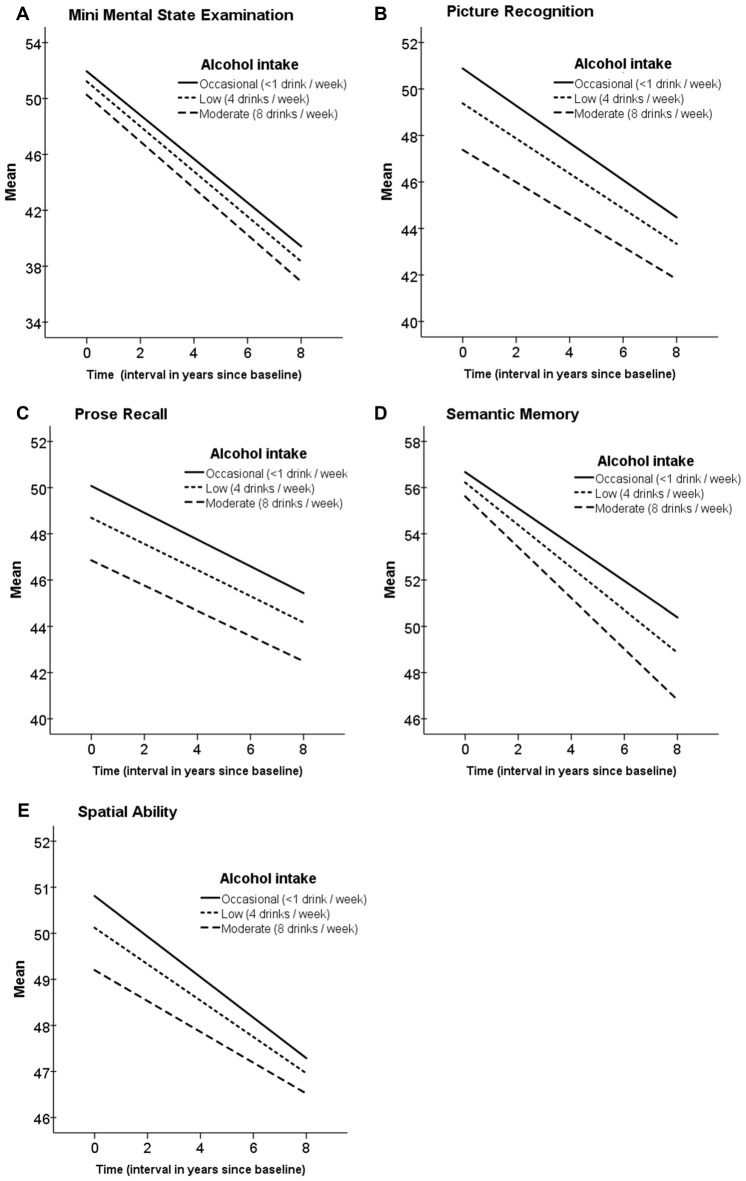
Trajectories of cognitive function at age 80+ in relation to alcohol intake in midlife. Estimated means of linear mixed models adjusted for age, education, gender and socioeconomic position. Time 0 is the time of the first wave of the cognitive testing. **(A)** Mini Mental State Examination; **(B)** Picture Recognition; **(C)** Prode Recall; **(D)** Semantic Memory; **(E)** Spatial Ability.

Alcohol consumption in midlife was low in general and lower in women as compared to men (see Figure 1). The results from the growth curve models are presented in Table 3 and illustrated in Figure 2. In general, the estimates of the effect of alcohol intake in midlife on level of cognitive performance in old age were all negative, ranging between −0.10 and −0.57 (see the estimates of Alcohol in Table 3). When inspecting the findings from Model 1, which was adjusted for demographic characteristics, it can be seen that higher alcohol consumption in midlife was linearly related to lower performance in all cognitive tests. These associations were statistically significant for the MMSE (*t*_(303)_ = 2.63) and the two long-term memory tasks, that is, picture recognition (*t*_(289)_ = 2.45) and prose recall (*t*_(299)_ = 2.13). In Model 2, which was additionally adjusted for health-related factors, there were only significant negative associations between alcohol consumption and picture recognition (*t*_(293)_ = 2.32).

**Table 3:** Parameter estimates for the Intercept, Slope and Alcohol intake across cognitive tests (only Alcohol consumers included, *N* = 305).

	MMSE			Picture Recognition			Prose Recall			Semantic Memory			Spatial Ability		
	Est.	*SE*	*p*	Est.	*SE*	*p*	Est.	*SE*	*p*	Est.	*SE*	*p*	Est.	*SE*	*p*
Model 1^a^
Intercept	52.11	1.19	<0.001	51.36	2.05	<0.001	50.29	2.04	<0.001	56.85	1.93	<0.001	51.03	2.24	<0.001
Slope	−3.12	0.35	<0.001	−1.63	0.29	<0.001	−1.17	0.21	<0.001	−1.48	0.17	<0.001	−0.91	0.19	<0.001
Alcohol	−0.34	0.13	0.009	−0.57	0.23	0.015	−0.48	0.23	0.034	−0.28	0.21	0.184	−0.29	0.26	0.254
Slope × Alcohol	−0.02	0.12	0.850	0.03	0.10	0.736	0.00	0.07	0.953	−0.09	0.06	0.126	−0.03	0.06	0.598
Model 2^b^
Intercept	60.90	2.95	<0.001	55.96	5.05	<0.001	60.83	5.17	<0.001	69.67	4.85	<0.001	67.15	5.26	<0.001
Slope	−3.08	0.35	<0.001	−1.63	0.29	<0.001	−1.14	0.21	<0.001	−1.48	0.17	<0.001	−0.91	0.18	<0.001
Alcohol	−0.24	0.13	0.058	−0.50	0.23	0.032	−0.37	0.23	0.108	−0.23	0.21	0.272	−0.10	0.25	0.672
Slope × Alcohol	−0.02	0.12	0.858	0.03	0.10	0.753	0.01	0.07	0.879	−0.09	0.06	0.135	−0.03	0.06	0.630

*Notes: Est. = Parameter estimator, SE = Standard error, p = p-value. Significance of these coefficients is determined with a t-statistic (df = 283–305). ^a^Adjusted for age, gender, education and socioeconomic position. ^b^Adjusted additionally for BMI in 1967, diabetes, vascular diseases and smoking*.

### Alcohol Intake in Relation to Cognitive Decline Across Time

In general, there was a significant aging-related decline across time in all cognitive tasks as reflected by the estimates of the Slope in Table 3. However, there was no additional decline in cognitive performance related to alcohol intake as can be seen by the non-significant estimates for the Slope × Alcohol terms in Models 1 and 2 in Table 3.

## Discussion

The aim of this study was to re-examine the association between alcohol intake and cognitive function in old age in while controlling the abstainer bias. First, it was found that the group of abstainers differed from the drinkers in several aspects relating to demographic- and health-related factors. Thereby, there was a clear abstainer bias in this study. Second, the main findings from this study, in which the abstainer bias was controlled, showed that there was a negative and not a positive effect of light alcohol intake on cognitive performance.

The findings from Model 1, which controlled for demographic factors only, showed negative association between alcohol intake reported in midlife and cognitive function 25-years later, such that more drinking was significantly related to lower scores in the MMSE, picture recognition, and prose recall. Further adjustments for health-related factors in Model 2 resulted in attenuated estimates for the alcohol associations for all the cognitive tests. This indicates that there is a covariation between the effect of alcohol and the health-related factors, such that a part of the negative effect of alcohol on cognitive performance may have been caused by poor health. Considering the selective effect of alcohol on the different cognitive abilities, it is well established that crystallized abilities are stable across the lifespan and relatively unaffected by normal ageing and diseases, in contrast to memory and fluid abilities. The differential findings across the cognitive tests in the present study are in line with this; the alcohol effects were seen in the episodic memory tests but not in semantic memory (a crystallized ability). Regarding the trajectories of cognitive performance across time, there was a significant decline in all the cognitive measures, however, there was no additional decline related to alcohol intake. Given the high age of this sample, many individuals can be considered to be in the terminal phase of life, resulting in terminal decline in cognitive performance, which may have overshadowed the negative effect of alcohol that was found in the levels of cognitive performance. The sizes of the estimates for the slopes, reflecting considerable ageing-related decline, support this notion.

Concerning how these findings should be interpreted it is clear that they are in line with many findings showing negative effects of acute alcohol on cognitive function (Matthews and Silvers, 2004; Mintzer, 2007; Fillmore, 2011; Dry et al., 2012). However, they contradict the conclusion that long-term light to moderate alcohol intake promotes cognitive health, an established finding, according to the literature. The obvious difference between the present study and almost all other studies on this matter is that the present study does not use the abstainers as a reference group. This was done in an attempt to deal with the abstainer bias, as the group of non-drinkers has in other studies been shown to have higher prevalence of a range of negative outcomes such as negative psychosocial characteristics, lower education and income, lacking access to health care and services, higher comorbid health conditions such as diabetes and hypertension, lower levels of mental well-being, and a higher CVD risk score (Leifman et al., 1995; Naimi et al., 2013). When inspecting the abstainer group in the present study, the same pattern emerged; the abstainer group had lower educational level, lower socioeconomic position, higher BMI in midlife, and higher prevalence of diabetes and hypertension. Thus, the findings from the present study are in line with the conclusion of Stockwell et al. (2016) who showed that when the abstainer bias was accounted for, the pattern of results describing the relationships between level of alcohol consumption and all-cause mortality was more consistent with a linear dose response than a J-shaped curve.

It should be noted that the participants in this study reported low intake and frequency of alcohol use; 37% did not drink at all and only 8% reported drinking one drink or more/day. This can be contrasted to considerably higher alcohol consumption reported by later born cohorts in Sweden; in a cohort of 75-year old Swedes born in 1930, 66% report drinking at least two times/week (Waern et al., 2014). Despite the low alcohol intake in the present study, there were clear negative effects on cognitive function. To exemplify, one unit of alcohol a week was reflected by −0.57 T-score units for picture recognition, whereas one unit a day would result in −3.99 T-score units, which corresponds to approximately 80% of a standard deviation of the test. This may not be a strong effect, but it should be kept in mind that recommendation for the upper “safe limit” for men is 14 units/week and 7 units/week for women (National Institute on Alcohol Abuse and Alcoholism, 2017). Given the results from the present study, these guidelines may potentially be too high.

There are a few methodological issues that need to be discussed. The low alcohol consumption reported in the present study may limit the external validity of the study as the findings may, in a strict way, only be generalized to low consumers. However, as the study analyzed the association by modeling dose-response, the effect of higher alcohol intake on cognitive function could be predicted by the models. Another methodological issue that should be considered, as in all studies on older populations, is the survival effect. The sample in this study was 80 years and older at the time of the first cognitive assessment which means that many individuals did not survive into this advanced age. Further, as high alcohol consumption is related to shorter survival there were no heavy drinkers in this sample, resulting in restricted range in the alcohol variable.

The main strengths of this study are related to the sampling, the cognitive testing and the longitudinal design. The study sample is a population-based sample that gives strength to the generalizability of the results. The cognitive assessments include several validated tests that capture specific domains of cognitive functioning. The longitudinal design, including cognitive assessments at five time-points, makes it possible to examine both level of cognitive performance and cognitive trajectories across time.

To summarize, the present study showed that higher alcohol consumption was related to lower cognitive test performance. Therefore, it is concluded that there are no beneficial effects of alcohol on cognitive function and clear negative effects on episodic memory. The results from this study indicate that the observed benefits of moderate alcohol intake for cognitive function might be solely due to comparisons to an inappropriate control group, a group that is biased towards poor health. These findings need to be replicated in younger old age cohorts and in cohorts who report higher alcohol intake. It is important to clarify the association between alcohol drinking and cognitive health, since initiating or increasing alcohol consumption is combined with multiple risks in many aspects of life.

## Author Contributions

The author has done all parts in this work and takes full responsibility for the data, the analyses and interpretation and the conduct of the research.

## Conflict of Interest Statement

The author declares that the research was conducted in the absence of any commercial or financial relationships that could be construed as a potential conflict of interest.
